# Identification of hub genes and regulatory networks in histologically unstable carotid atherosclerotic plaque by bioinformatics analysis

**DOI:** 10.1186/s12920-022-01257-1

**Published:** 2022-06-30

**Authors:** Julong Guo, Yachan Ning, Zhixiang Su, Lianrui Guo, Yongquan Gu

**Affiliations:** 1 Department of Vascular Surgery, Xuanwu Hospital, Capital Medical University, No. 45 Changchun Street, Xicheng District, Beijing, 100053 China; 2 Department of Intensive Care Medicine, Xuanwu Hospital, Capital Medical University, Beijing, China

**Keywords:** Atherosclerosis, Unstable carotid artery plaque, Bioinformatics, Potential biomarker, Therapeutic target

## Abstract

**Objective:**

This study identified underlying genetic molecules associated with histologically unstable carotid atherosclerotic plaques through bioinformatics analysis that may be potential biomarkers and therapeutic targets.

**Methods:**

Three transcriptome datasets (GSE41571, GSE120521 and E-MTAB-2055) and one non-coding RNA dataset (GSE111794) that met histological grouping criteria of unstable plaque were downloaded. The common differentially expressed genes (co-DEGs) of unstable plaques identified from three mRNA datasets were annotated by Gene Ontology (GO) and Kyoto Encyclopedia of Genes and Genomics (KEGG). A protein–protein interaction (PPI) network was constructed to present the interaction between co-DEGs and screen out hub genes. MiRNet database and GSE111794 dataset were used to identify the miRNAs targeting hub genes. Associated transcription factors (TFs) and drugs were also predicted. These predicted results were used to construct miRNA/TFs-hub gene and drug-hub gene regulatory networks.

**Results:**

A total of 105 co-DEGs were identified, including 42 up-regulated genes and 63 down-regulated genes, which were mainly enriched in collagen-containing extracellular matrix, focal adhesion, actin filament bundle, chemokine signaling pathway and regulates of actin cytoskeleton. Ten hub genes (up-regulated: HCK, C1QC, CD14, FCER1G, LCP1 and RAC2; down-regulated: TPM1, MYH10, PLS3 and FMOD) were screened. HCK and RAC2 were involved in chemokine signaling pathway, MYH10 and RAC2 were involved in regulation of actin cytoskeleton. We also predicted 12 miRNAs, top5 TFs and 25 drugs targeting hub genes. In the miRNA/TF-hub gene regulatory network, PLS3 was the most connected hub genes and was targeted by six miRNAs and all five screened TFs. In the drug-hub gene regulatory network, HCK was targeted by 20 drugs including 10 inhibitors.

**Conclusions:**

We screened 10 hub genes and predicted miRNAs and TFs targeting them. These molecules may play a crucial role in the progression of histologically unstable carotid plaques and serve as potential biomarkers and therapeutic targets.

**Supplementary Information:**

The online version contains supplementary material available at 10.1186/s12920-022-01257-1.

## Introduction

Carotid artery stenosis, most commonly caused by atherosclerosis, is a main reason of ischemic stroke [1]. However, there is increasing evidence that in addition to atherosclerotic stenosis, unstable plaques also play an important role in promoting symptomatic stroke [2, 3]. In advanced atherosclerosis, plaques may have a large necrotic lipid core, a weak fibrous cap, intraplaque neovascularization and hemorrhage. These histological changes make the plaque unstable and prone to rupture, which is more likely to result in thrombosis and embolization of plaque material, thereby leading to vascular occlusion and subsequent ischemic stroke [3, 4]. Moreover, unstable plaques cause more severe neurological damage in patients with acute cerebral infarction than stable plaques [5]. Therefore, clarifying the pathogenesis associated with unstable plaque formation is significant and may reveal potential biomarkers and therapeutic targets.

With the further research of the pathogenesis, bioinformatics analysis has become an essential technique for discovering genetic alteration in various diseases. By analyzing microarray or sequencing data, the differentially expressed genes (DEGs) of unstable carotid plaques can be found. The functions and pathways involved in DEGs were then predicted by functional enrichment analysis. Further construction of protein–protein interaction (PPI) network can understand their interactions and screen out the hub genes related to unstable carotid plaques. Finally, prediction of miRNAs, transcription factors (TFs) and drugs targeting hub genes can make the pathogenesis network more comprehensive.

Several unstable carotid plaque datasets based on clinical and/or histological criteria have been published. In fact, unstable carotid plaques identified by clinical criteria alone, also known as symptomatic carotid stenosis, do not necessarily meet the histological criteria [6]. Some studies have pooled the datasets of unstable plaques that meet symptomatic or histological criteria, which may produce inaccurate results. In this study, three transcriptome datasets and one non-coding RNA dataset met histological criteria of unstable carotid plaques were selected from the Gene Expression Omnibus (GEO, http://www.ncbi.nlm.nih.gov/geo/) database and the ArrayExpress (https://www.ebi.ac.uk/arrayexpress/) database for bioinformatics analysis, hoping to provide more insights into unstable carotid plaques.

## Methods

**Fig. 1:**
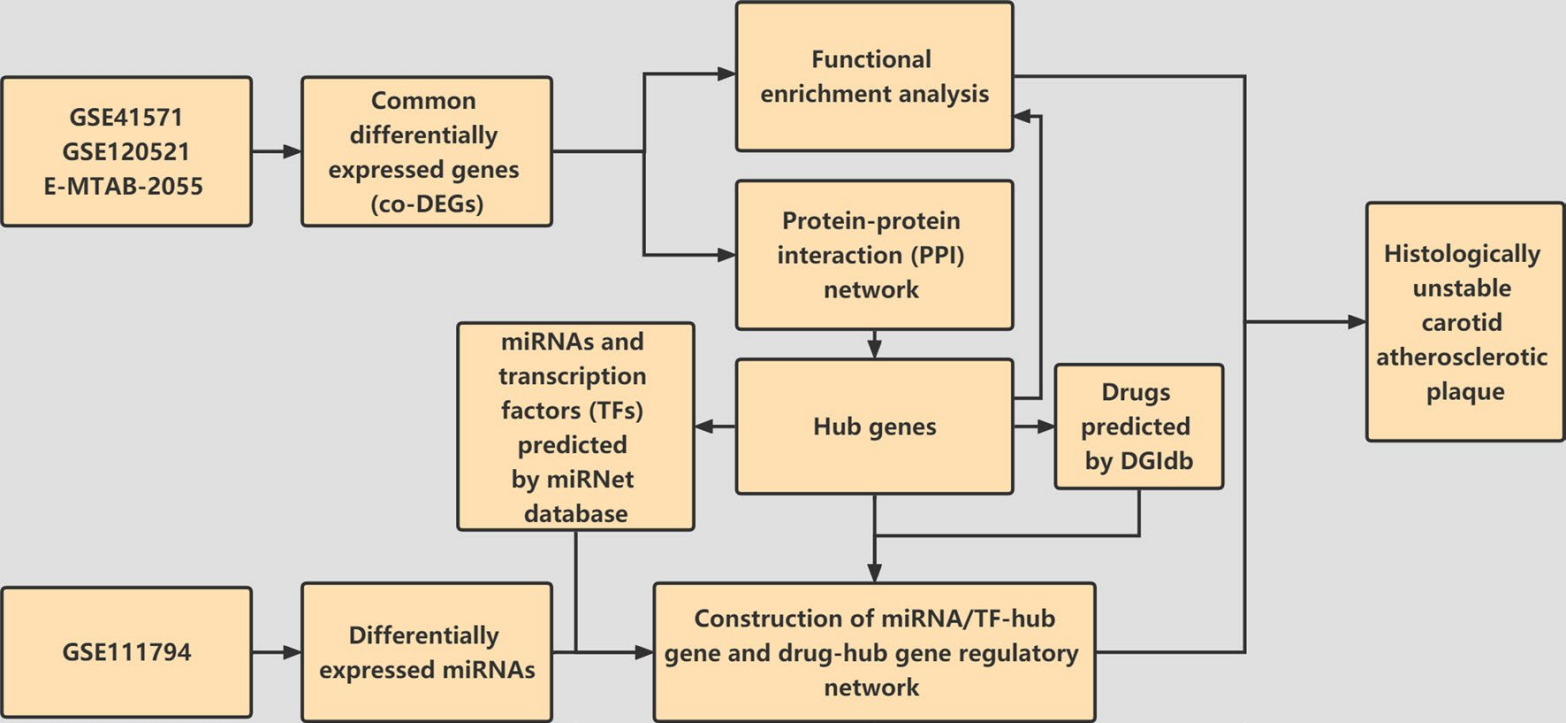
Flow chart

The study flow was shown in Fig. 1.

### Data source

Three gene expression profiles were downloaded. The matrix data of GSE41571 (microarray) and the Fragments Per Kilobase Million (FPKM) data of GSE120521 (RNA-sequencing) were obtained from the GEO database, and the processed data of E-MTAB-2055 (microarray) was downloaded from the ArrayExpress database. GSE41571, including 5 ruptured plaques and 6 stable plaques, was assayed at the platform of GPL570. GSE120521, containing 4 unstable plaques and 4 stable plaques, was measured at the platform of GPL16791. The platform of A-MEXP-931 was used by E-MTAB-2055 which included 25 ruptured plaques and 22 stable plaques. GSE111794 is a microRNA expression profile downloaded from the GEO database. It used GPL16384 platform to assay 9 unstable and 9 stable plaques. The local research ethics committee authorized construction of these three datasets.

### Identification of DEGs

NetworkAnalyst [7] (https://www.networkanalyst.ca/), a comprehensive gene-centric platform supporting gene expression profiling, was used to identify the DEGs of GSE41571 and GSE120521. The DEGs of E-MTAB-2055 were screened directly from its processed data. DEGs were defined as genes with *p*-value < 0.05 and |log fold change (FC)|> 1. The screening results were visualized using volcano maps drawn by GraphPad Prism 9.0.0. An online drawing tool (http://bioinformatics.psb.ugent.be/webtools/Venn/) was used to select the common DEGs (co-DEGs) of three datasets and draw a Venn diagram.

### Functional enrichment analysis

To further investigate the potential functions of these co-DEGs, the list of co-DEGs was uploaded to Metascape [8] (https://metascape.org/) for Gene Ontology (GO) [9] and Kyoto Encyclopedia of Genes and Genomes (KEGG) [10] pathway enrichment analysis. Metascape is a web-based portal leveraging over 40 independent knowledgebases to provide a comprehensive gene list annotation and analysis resource for users [8]. Cellular compartments (CC), biological processes (BP), molecular function (MF) and KEGG pathway were selected for analysis. The threshold for P-value was set at 0.05 and the minimum enrichment score was 1.5. The visualization results were generated by Metascape and GraphPad Prism 9.0.0.

### PPI network and hub gene identification

The Search Tool for the Retrieval of Interacting Genes [11] (STRING, v11.5, https://cn.string-db.org/) is a database of known and predicted PPIs. After uploading the co-DEGs list to the STRING database, PPI results were obtained with required interaction score > 0.4. Cytoscape (v3.9.0) [12] was then used to construct a visual PPI network and identify the hub genes. In order to avoid inaccurate screening results caused by using a single rank method, maximal clique centrality (MCC), maximum neighborhood component (MNC), node connect degree (Degree), edge percolated component (EPC), closeness and radiality rank methods were used for screening top 10 genes, respectively. All of these rank methods are included in Cytoscape’s application named Cytohubba (v0.1) [13]. Their results were integrated, then the genes with the top 10 frequencies were identified as the hub genes.

### Prediction of miRNA/TF‑hub gene regulatory network

The miRNAs targeting hub genes were predicted using miRNet [14] (v2.0, https://www.mirnet.ca/), which is a miRNA-centric network visual analytics platform. The miRNA-hub gene data were collected from the well-annotated database miRTarBase v8.0 [15], DIANA-TarBase v8.0 [16], and miRecords [17]. The differentially expressed miRNAs of GSE111794 were analyzed using GEO2R, and then intersected with the predicted results of miRNet 2.0 to identify meaningful miRNA. TFs were also predicted in miRNet, using JASPAR database[18] resources. The top5 TFs were selected to construct the regulatory network. Finally, we visualized the miRNA/TF-hub gene regulatory network using Cytoscape 3.9.0.

### Prediction of drug-hub gene regulatory network

The drugs targeting all hub genes were predicted using the Drug Gene Interaction Database [19] (DGIdb, v4.2.0, https://dgidb.genome.wustl.edu/). DGIdb used a combination of expert curation and text-mining to mine drug-gene interactions from DrugBank [20], PharmGKB [21], Drug Target Commons [22] and others. The Cytoscape3.9.0 was used to construct the drug–gene regulatory network.

## Results

### Identification of DEGs

**Fig. 2:**
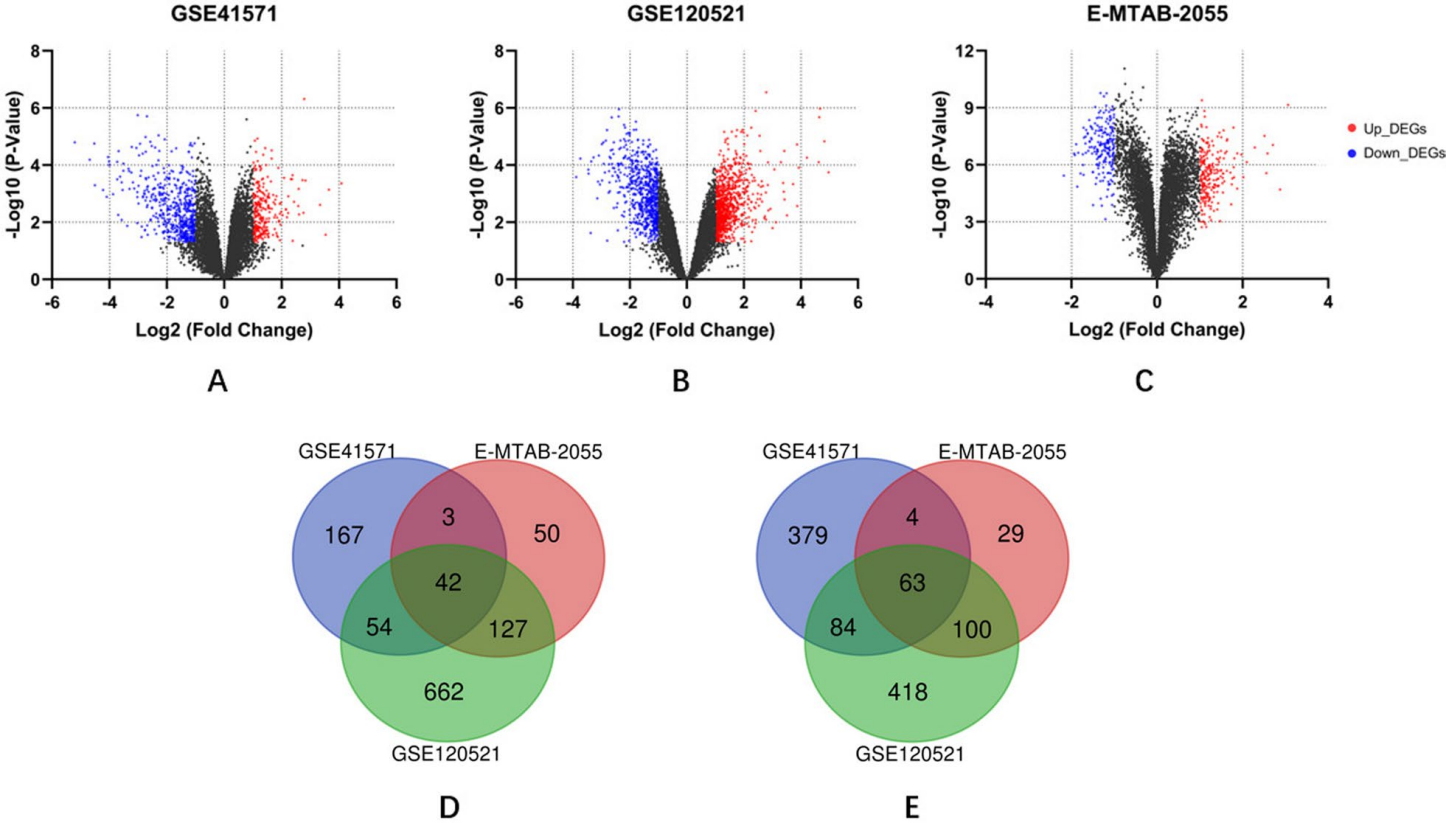
Identification of DEGs. **A**: Volcano plot of GSE41571; **B**: Volcano plot of GSE120521; **C**: Volcano plot of E-MTAB-2055; **D**: Venn diagram of up-regulated DEGs; **E**: Venn diagram of down-regulated DEGs

DEGs between unstable and stable carotid plaques were identified. Seven hundred and ninety-six DEGs were found in GSE41571, including 266 up-regulated and 530 down-regulated DEGs (Fig. 2A). There were 796 DEGs in GSE41571, including 266 up-regulated and 530 down-regulated DEGs (Fig. 2B). And we obtained 418 DEGs in E-MTAB-2055, including 222 up-regulated and 196 down-regulated DEGs (Fig. 2C). After integration, a total of 105 co-DEGs including 42 up-regulated and 63 down-regulated DEGs overlapped in three datasets (Fig. 2D, E).

### GO and KEGG enrichment analysis

**Fig. 3:**
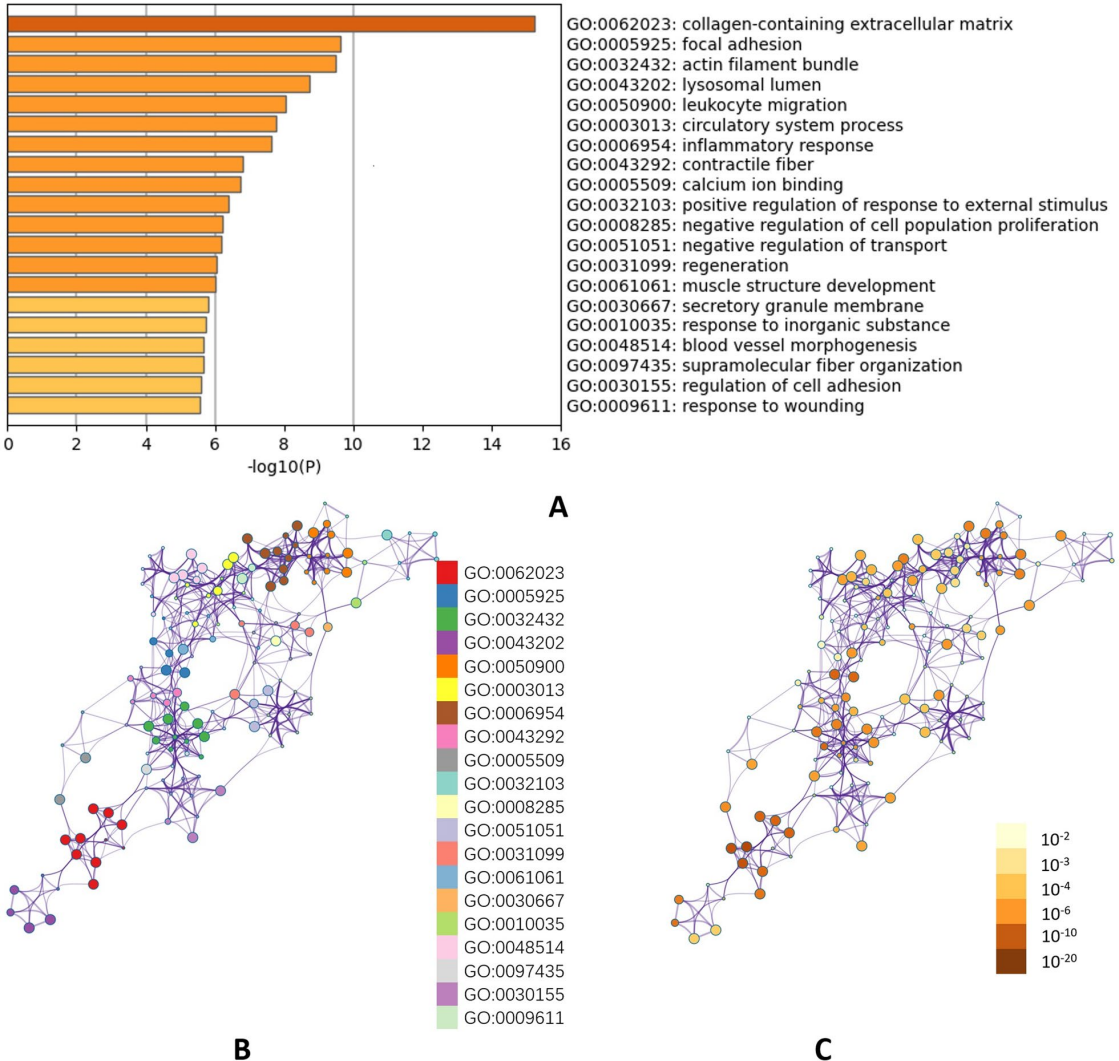
GO and KEGG Enrichment Analyses. (**A**): Bar graph of enriched terms across input gene lists, colored by *p*-values. **B**, **C**: Network of enriched terms: (**B**) colored by cluster ID, where nodes that share the same cluster ID are typically close to each other; (**C**) colored by *p*-value, where terms containing more genes tend to have a more significant *p*-value

**Fig. 4:**
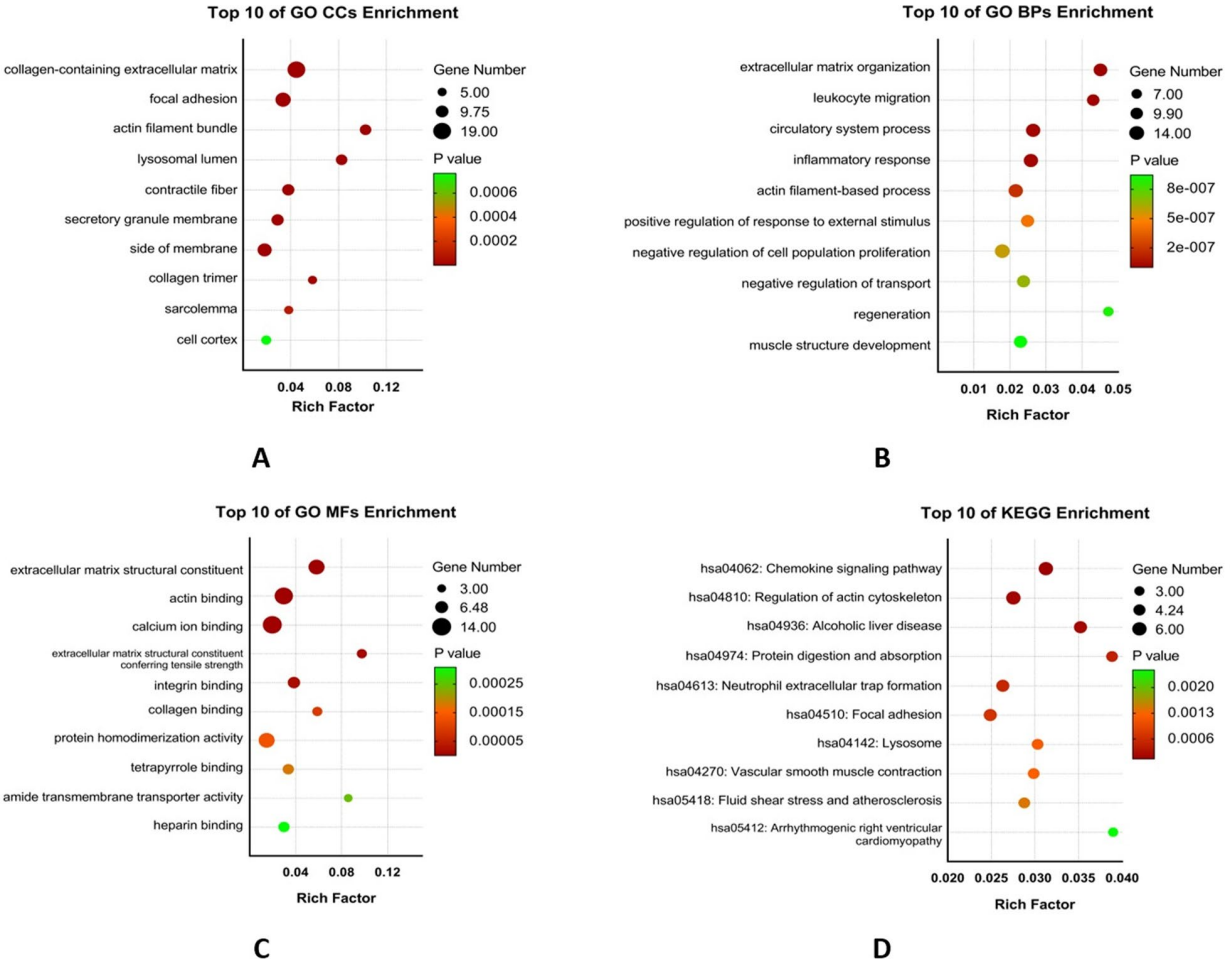
Bubble diagram of the functional enrichment analysis of DEGs. **A**: biological processes of GO enrichment; **B**: cellular compartments of GO enrichment; **C**: molecular function of GO enrichment; **D**: KEGG pathway analysis

All 105 co-DEGs were uploaded to Metascape database for functional enrichment analysis. The first 20 representative enriched terms (one for each cluster) are shown in Fig. 3A and Additional file 1: Table S1. To further capture the relationships between these terms, Metascape presented a network where terms with a similarity > 0.3 are connected by edges (Fig. 3B, C). The top 3 enriched terms were collagen-containing extracellular matrix, focal adhesion and actin filament bundle, all of which belong to CC (Figs. 3A, 4A). As summarized in Table 1, co-DEGs were mostly enriched in BP of extracellular matrix organization, leukocyte migration and circulatory system (Fig. 4B). For MF, these co-DEGs were particularly enriched in extracellular matrix structural constituent, actin binding and calcium ion binding (Fig. 4C). KEGG pathway analysis revealed co-DEGs were primarily enriched in chemokine signaling pathway and regulation of actin cytoskeleton (Fig. 4D).

**Table 1:** Top 3 terms of GO and top 2 terms of KEGG pathway functional enrichment analysis of each category

Category	Term	Log P	Count	Hub genes
GO CCs	GO:0062023: collagen-containing extracellular matrix	− 15.26	19	C1QC, FMOD
GO CCs	GO:0005925: focal adhesion	− 9.64	14	HCK, LCP1, RAC2
GO CCs	GO:0032432: actin filament bundle	− 9.51	8	LCP1, MYH10, PLS3, TPM1
GO BPs	GO:0030198: extracellular matrix organization	− 9.8	12	FMOD, LCP1
GO BPs	GO:0050900: leukocyte migration	− 8.05	10	FCER1G, HCK
GO BPs	GO:0003013: circulatory system process	− 7.76	13	TPM1
GO MFs	GO:0005201: extracellular matrix structural constituent	− 9.3	10	FMOD
GO MFs	GO:0003779: actin binding	− 8.35	13	LCP1, MYH10, PLS3, TPM1
GO MFs	GO:0005509: calcium ion binding	− 6.73	14	LCP1, PLS3
KEGG	hsa04062: Chemokine signaling pathway	− 4.24	6	HCK, RAC2
KEGG	hsa04810: Regulation of actin cytoskeleton	− 4	6	MYH10, RAC2

*GO* Gene Ontology, *KEGG* Kyoto Encyclopedia of Genes and Genomes, *CC* Cellular compartment, *BP* Biological processes, *MF* Molecular function

### PPI network and hub gene identification

**Fig. 5:**
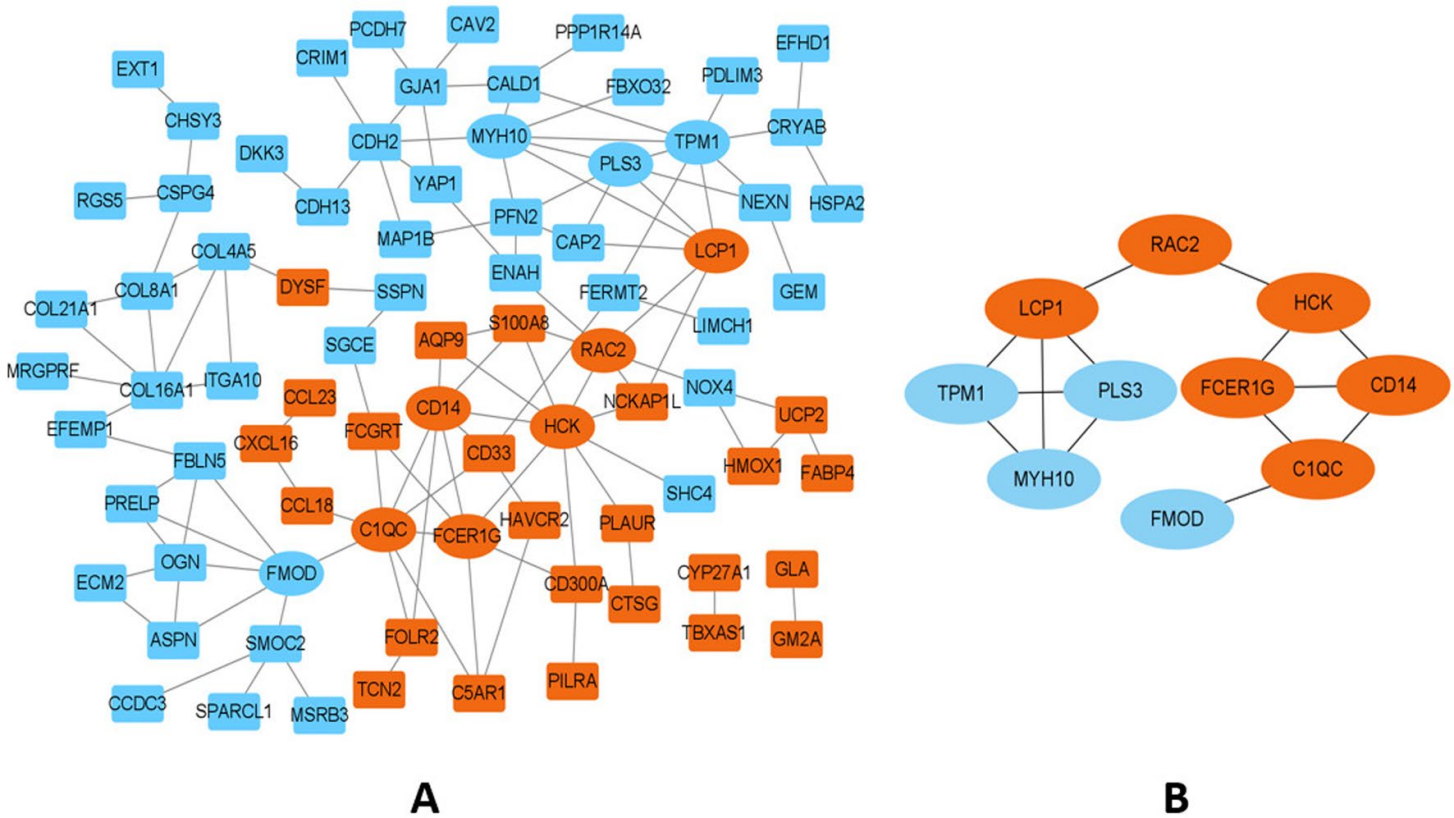
Protein–protein interaction network of co-DEGs (**A**) and the hub genes (**B**). Orange represents the up-regulated co-DEGs. Cyan represents down-regulated co-DEGs. Rectangles represent co-DEGs except for hub genes. Circles represent hub genes

A PPI network with 105 nodes and 116 interacting pairs was constructed (Fig. 5A). The top10 DEGs were identified as hub genes by six rank methods of Cytoscape Cytohubba (Table 2). Among them, four genes (HCK, C1QC, TPM1 and CD14) were selected in all six methods, and FMOD and PLS3 with the lowest frequency appeared in the results of four methods. The identified 10 hub genes included 6 up-regulated genes (HCK C1QC, CD14, FCER1G, LCP1 and RAC2) and 4 down-regulated genes (TPM1, MYH10, FMOD and PLS3) (Fig. 5B). All of the hub genes were present in the top enriched functional terms and may play critical roles in atherosclerotic carotid unstable plaques. Furthermore, HCK and RAC2 were enriched in chemokine signaling pathway, MYH10 and RAC2 were enriched in regulation of actin cytoskeleton.

**Table 2:** Top 10 DEGs screened by different rank methods and the result of hub genes

MCC	MNC	Degree	EPC	Closeness	Radiality	Hub genes
HCK	HCK	HCK	HCK	C1QC	C1QC	HCK
PLS3	CD14	C1QC	CD14	HCK	CD33	C1QC
CD14	PLS3	TPM1	RAC2	TPM1	CD14	TPM1
TPM1	C1QC	CD14	LCP1	CD14	HCK	CD14
MYH10	FCER1G	MYH10	C1QC	RAC2	FCER1G	MYH10
C1QC	TPM1	PLS3	FCER1G	FCER1G	FERMT2	RAC2
LCP1	MYH10	CDH2	MYH10	MYH10	RAC2	FCER1G
FCER1G	OGN	LCP1	TPM1	LCP1	S100A8	LCP1
FMOD	LCP1	FMOD	PLS3	CD33	TPM1	FMOD
OGN	RAC2	RAC2	S100A8	FMOD	FMOD	PLS3

*MCC* maximal clique centrality, *MNC* maximum neighborhood component, Degree: node connect degree, *EPC* edge percolated component

### miRNA/TF‑hub gene regulatory network

**Fig. 6:**
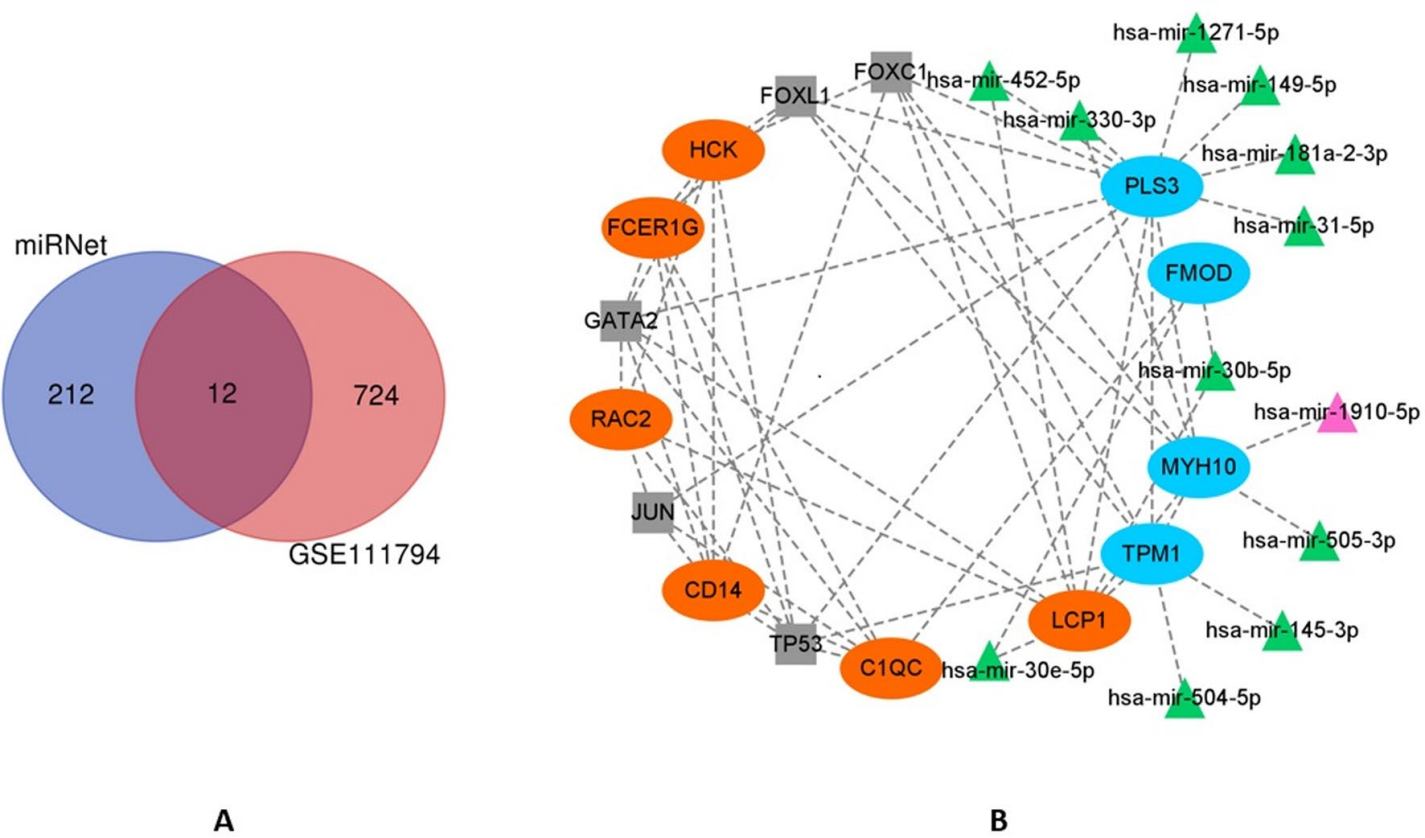
Prediction of miRNA/TF‑hub gene regulatory network. **A**: Venn diagram of miRNAs predicted by miRNet and differentially expressed in GSE111794; **B**: miRNA/TF‑hub gene regulatory network. Orange circles represent up-regulated hub genes. Cyan circles represent down-regulated hub genes. Pink triangles represent up-regulated miRNAs. Green triangles represent down-regulated miRNAs. Gray squares represent transcription factors

Two hundred and twenty-four miRNAs targeting hub genes were predicted using miRNet. There were 736 differentially expressed miRNAs in the GSE111794 dataset, among which 12 miRNAs overlapped with the miRNet results (Fig. 6A), including 1 up-regulated and 11 down-regulated miRNAs targeting five hub genes (TPM1, MYH10, PLS3, LCP1 and FMOD). The Top5 TFs targeting hub genes were predicted to be GATA2, TP53, FOXC1, FOXL1 and JUN. Then a miRNA/TF-hub gene regulatory network was constructed (Fig. 6B). PLS3 was the most targeted hub genes. PLS3 was targeted by six miRNAs and all five screened TFs.

### Drug-hub gene regulatory network

**Fig. 7:**
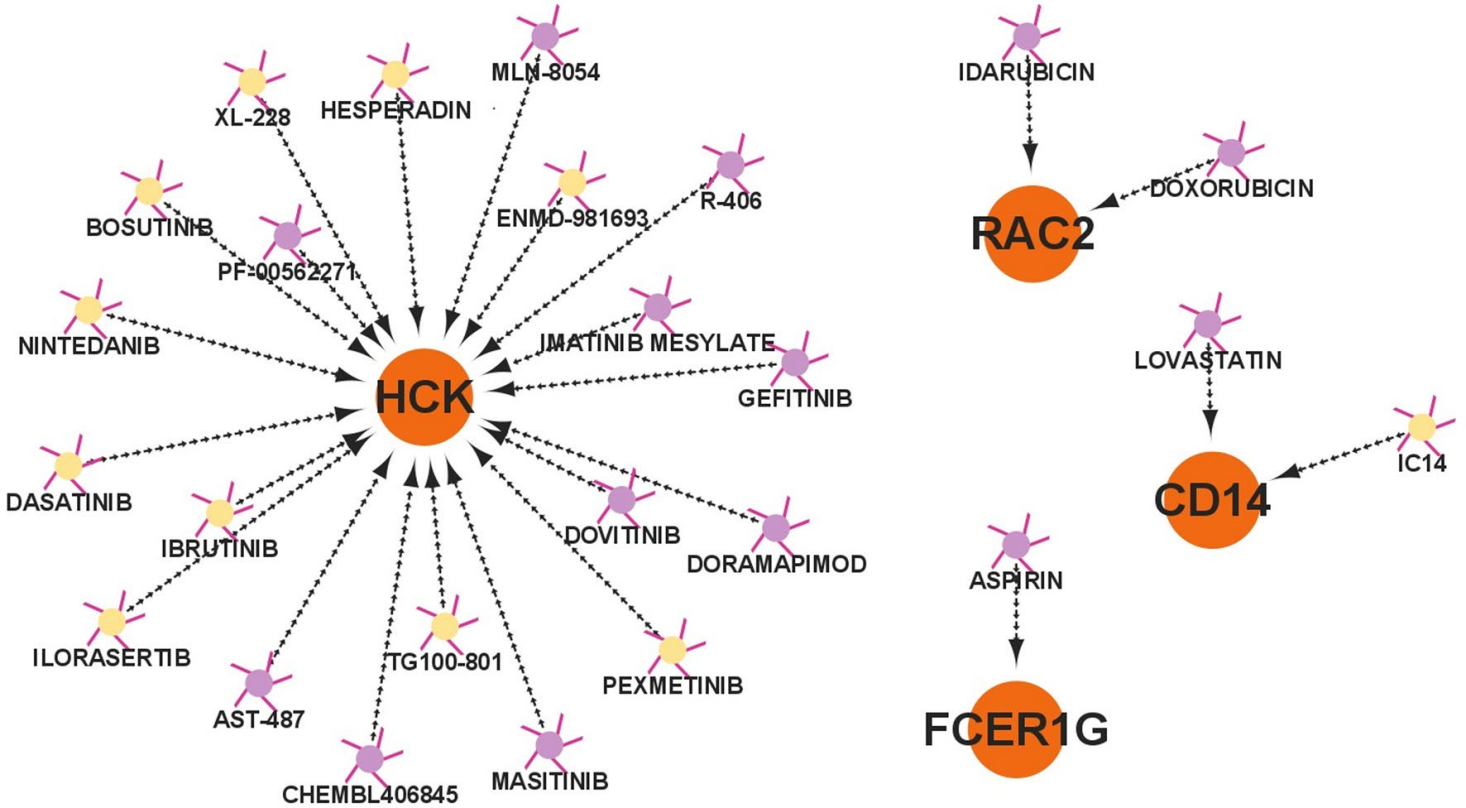
Drug-hub gene regulatory network. Orange circles represent up-regulated hub genes. Yellow dots represent inhibitor drugs. Purple dots represent drugs without specified interaction type

As shown in Fig. 7, 25 potential drugs were found for 4 up-regulated hub genes using the DGIdb database. Among them, HCK was targeted by 20 drugs including 10 inhibitors. Drugs targeted the other 6 hub genes were not predicted.

## Discussion

We screened 105 co-DEGs from three data sets, including 42 up-regulated and 63 down-regulated genes. Through enrichment analysis of co-DEGs, we identified biological processes, cellular compartments and molecular functions associated with unstable carotid plaques. Atherosclerosis is associated with uncontrolled extracellular matrix (ECM) remodeling [23]. Reduced collagen content in ECM is a typical feature of unstable plaques [24]. Apolipoprotein E-deficient mice expressing collagenase-resistant collagen-I or lacking matrix metalloproteinase-13 (MMP-13/collagenase-3) can obtain collagen accumulation resulting in more stable plaques [25, 26]. Focal adhesions (FAs) are key junctions between cells and ECM and points of termination of actin filaments. FAs play an important role in tissue remodeling, integrity and homeostasis. The changes of FA constituents, such as the low expression of Talin and Vinculin, can affect the tissue remodeling and healing capabilities, which promotes the development of vulnerable plaques [27]. Actin filament bundles are the assemblies of actin filaments that participate in the regulation of endothelial cell (EC) adhesion to adjacent cells and matrix. During the development of arteriosclerosis, actin bundles change dynamically, and eventually the decrease or even disappearance of central actin microfilaments leads to dysfunction of cell–matrix adhesion [28]. In addition, calcification has been found to affect plaque stability. Common in unstable plaques are microcalcifications that originate from matrix vesicles rich in calcium-binding protein [29, 30]. The infiltration and activation of leukocytes within lesions also contribute to plaque rupture. For example, invariant natural killer T (iNKT) cells can promote rupture by activating inflammatory cells and up-regulating MMP-2 in vascular tissue [31]. In our study, the top2 enriched pathways are chemokine signaling pathway and regulation of actin cytoskeleton, each of which has been mentioned in previous studies to be involved in the progression of atherosclerotic plaque [32–34].

After constructing the PPI network, we identified 10 hub genes of unstable carotid plaque, including 6 up-regulated genes (HCK, C1QC, CD14, FCER1G, LCP1 and RAC2) and 4 down-regulated genes (TPM1, MYH10, PLS3 and FMOD). MiRNAs, TFs and drugs targeting these hub genes were also predicted.

HCK, a member of the SRC family of cytoplasmic tyrosine kinases (SFKs), is confined to the hematopoietic system, such as cells of myeloid and B lymphocyte lineages [35]. HCK promotes monocyte/macrophage intravasation by participating in a broad spectrum of processes including monocyte/macrophage proliferation, migration and endothelial adhesion, which is an essential mechanism of atherosclerosis involving a series of signaling pathways initiated by integrin, immune and growth factors, Fcy and chemokine receptors [36]. In addition, HCK can facilitate ECM degradation by phosphorylating the Wiskott-Aldrich syndrome protein (WASP) [37], which may contribute to plaque instability. However, Medina et al. indicated that atherosclerotic plaques showed reduced size in HCK/FGR double knockout mice but presented vulnerable phenotype characterized by necrotic core expansion, and significant reductions in collagen and fibrous cap thickness [36]. This may be due to a more complex response caused by the complete absence of HCK or the simultaneous deletion of FGR which is another member of SFKs, but it deserves further study. RAC2, a member of the Rho GTPases, is only found in cells of myeloid origin and is a crucial factor in leukocyte chemotaxis, as well as involved in the regulation of actin and microtubule cytoskeletal dynamics and adhesion [38]. RAC2 has been demonstrated to participate in reactive oxygen species (ROS) production [39], which induces the release of MMPs leading to plaque vulnerability by degrading the fibrous wall of atheromatous plaques and the basal membrane of endothelial cells [40]. MYH10 encodes a constituent protein of non-muscle myosin II, which is an actin-dependent motor protein and plays fundamental roles in cell adhesion and migration [41]. Kim et al. suggested that structural changes in the actomyosin network and defective ECM remodeling due to MYH10 deficiency contribute to the pathogenesis of emphysema [42], which may also contribute to unstable atherosclerotic plaques.

The other 7 hub genes were not enriched in the top 2 KEGG pathways. C1QC encodes the C-chain polypeptide of serum complement subcomponent C1q, a deficiency of which is associated with lupus erythematosus and glomerulonephritis. Lubbers et al. demonstrated that C1q can induce changes in ECM collagen expression [43]. C1QC was thought to regulate immune-competent cells involved in the progression of atherosclerosis [44]. CD14 is a differentiated antigen preferentially expressed on monocytes/macrophages and associated with inflammation and immune response. Inflammation can cause plaque disruption through processes such as endothelial cell death and activation of MMPs [45], but the role of CD14 needs to be further elucidated. It was found that the increase of CD14 + monocytes in coronary atherosclerosis patients was significantly correlated with the severity [46]. FCER1G is involved in encoding high affinity immunoglobulin epsilon receptors and has repeatedly been found to be overexpressed in the progression of atherosclerosis and unstable plaques. Although LCP1 (also named PLS2) and PLS3 are both actin binding proteins, LCP1 is overexpressed in unstable plaques, while PLS3 is underexpressed. LCP1 is essential for the degradation of ECM by macrophages. When LCP1 was inhibited by nanobodies, actin turnover was hampered and matrix degradation was significantly decreased [47]. PLS3 deficiency has been reported to cause osteoporosis and neurodegeneration [48]. TPM1 is a member of the tropomyosin family. Simoneau et al. proposed that TPM1 is required to maintain the endothelial barrier integrity and demonstrated that phosphorylation at Ser283 of TPM1 protects against oxidative stress-related endothelial barrier dysfunction [49]. FMOD encodes fibromodulin. In arteriosclerosis lesions, FMOD has stimulatory or stabilizing effects on collagen, smooth muscle cell proliferation, plaque lipids, inflammatory and proinflammatory cytokines [50].

In present study, we predicted 12 miRNAs that might be associated with unstable carotid plaques, several of which have been conducted the related studies. For example, mir-1910-5p was found to be highly expressed under oxidative stress [51], so it may be involved in ROS induced plaque destruction. Ye et al. demonstrated that lncRNA myocardial infarction associated transcript (MIAT) regulates the size of atherosclerotic necrotic core through the sponging mir-149-5p, while large necrotic core is a marker of unstable plaques [52]. A circulating microRNAs study observed that plasma mir-30e-5p was positively correlated with the volume of necrotic core in coronary plaques [53], but we found that mir-30e-5p was low expressed in unstable plaque samples. Whether this conflict is caused by different sample types or other reasons still needs further exploration. In addition, another study suggested that circulating mir-330-3p can be used to distinguish the plaque phenotype in patients with ST-segment elevation myocardial infarction [54]. TFs can affect gene expression and may play a role in unstable plaques. Therefore, we predicted the top five suspected TFs to improve the regulatory network of unstable plaques.

Several related drugs were predicted, including ten and one inhibitors of HCK and CD14. These drugs may be able to prevent or delay the progression of atherosclerotic plaque rupture. The eleven predicted inhibitors are mainly kinase inhibitors, which have been widely used in the treatment of malignant tumors. Several of them, such as nintedanib and bosutinib, have also been shown to ameliorate the development of atherosclerosis [55, 56].

Some limitations exist in our study. First of all, our analysis was based on four datasets from public data, whose grouping criteria may differ slightly, even though they all meet the histological criteria. In addition, only one miRNA dataset was used without cross-validation of other datasets, which may cause bias. Finally, we predicted the relevant molecules only by bioinformatic analysis, the true and complete regulatory mechanisms need to be verified by further in-vivo and in-vitro experiments.

## Conclusions

In conclusion, the rupture of carotid atherosclerotic plaque involves extremely complex molecular mechanisms and regulatory networks. Bioinformatics analysis was used to identify 105 DEGs that are mainly enriched in the chemokine signaling pathway and the regulation of actin cytoskeleton pathway to participate in the formation of histologically unstable carotid atherosclerotic plaques. We screened 10 hub genes and predicted miRNAs and TFs targeting them to construct the regulatory network. These molecules may play a crucial role in the progression of unstable carotid plaques and serve as potential biomarkers and therapeutic targets.

## Supplementary Information

**Additional file 1: Table S1**. Top 20 terms (one per cluster) of functional enrichment analysis.

## Data Availability

Publicly available datasets were analyzed in this study. This data can be found here: https://www.ncbi.nlm.nih.gov/geo/; https://www.ebi.ac.uk/arrayexpress/.

## Abbreviations

DEG: Differentially expressed gene
PPI: Protein–protein interaction
TF: Transcription factor
GEO: Gene Expression Omnibus
FPKM: Fragments per kilobase million
FC: Fold change
GO: Gene ontology
KEGG: Kyoto Encyclopedia of Genes and Genomes
CC: Cellular compartments
BP: Biological processes
MF: Molecular function
STRING: The Search Tool for the Retrieval of Interacting Genes
MCC: Maximal clique centrality
MNC: Maximum neighborhood component
EPC: Edge percolated component
DGIdb: The Drug Gene Interaction Database
ECM: Extracellular matrix
MMP: Matrix metalloproteinase
FA: Focal adhesion
EC: Endothelial cell
SFKs: The SRC family of cytoplasmic tyrosine kinases
WASP: Wiskott–Aldrich syndrome protein
ROS: Reactive oxygen species
MIAT: Myocardial infarction associated transcript
