# Supplementary material for: Identification of hub genes and regulatory networks in histologically unstable carotid atherosclerotic plaque by bioinformatics analysis
Source: BMC Med Genomics. 2022 Jun 30;15:145. doi: 10.1186/s12920-022-01257-1 (PMC9245266; doi:10.1186/s12920-022-01257-1)
Supplement: Supplementary file 1 — Additional file 1: Table S1. Top 20 terms (one per cluster) of functional enrichment analysis. [file 12920_2022_1257_MOESM1_ESM.docx]

Supplemental table 1. Top 20 terms (one per cluster) of functional enrichment analysis.

| Category | Description | LogP | count | gene symbol |
| --- | --- | --- | --- | --- |
| GO CC | GO:0062023 collagen-containing extracellular matrix | -15.26 | 19 | C1QC, CDH2, CDH13, COL4A5, COL8A1, COL16A1, CSPG4, CTSG, ECM2, EFEMP1, FMOD, OGN, PRELP, S100A8, SPARCL1, FBLN5, ASPN, SMOC2, COL21A1 |
| GO CC | GO:0005925 focal adhesion | -9.64 | 14 | CAV2, CDH2, CDH13, CSPG4, CSRP2, GJA1, HCK, LCP1, PLAUR, RAC2, FERMT2, NOX4, ENAH, NEXN |
| GO CC | GO:0032432 actin filament bundle | -9.51 | 8 | CRYAB, LCP1, MYH10, PLS3, TPM1, FERMT2, LIMCH1, PDLIM3 |
| GO CC | GO:0043202 lysosomal lumen | -8.74 | 8 | ACP2, CSPG4, FMOD, GLA, GM2A, OGN, PRELP, TCN2 |
| GO BP | GO:0050900 leukocyte migration | -8.05 | 10 | C5AR1, CTSG, EXT1, FCER1G, HCK, NCKAP1L, S100A8, CCL18, CCL23, CXCL16 |
| GO BP | GO:0003013 circulatory system process | -7.76 | 13 | ADM, CTSG, EXT1, FOXC1, GJA1, HMOX1, PLN, TBXAS1, TPM1, YAP1, FERMT2, NOX4, SLC24A3 |
| GO BP | GO:0006954 inflammatory response | -7.64 | 13 | ADM, C5AR1, CD14, EXT1, FOLR2, HCK, HMOX1, S100A8, CCL18, CCL23, AOC3, NOX4, HAVCR2 |
| GO CC | GO:0043292 contractile fiber | -6.81 | 9 | CALD1, CRYAB, CSRP2, GJA1, TPM1, FERMT2, PDLIM3, NEXN, FBXO32 |
| GO MF | GO:0005509 calcium ion binding | -6.73 | 14 | CDH2, CDH13, EFEMP1, LCP1, PCDH7, PLS3, S100A8, DYSF, SPARCL1, AOC3, FBLN5, ASPN, SMOC2, EFHD1 |
| GO BP | GO:0032103 positive regulation of response to external stimulus | -6.38 | 11 | C5AR1, CDH13, FABP4, HCK, NCKAP1L, MAPK13, RAC2, S100A8, PLA2G7, SMOC2, HAVCR2 |
| GO BP | GO:0008285 negative regulation of cell population proliferation | -6.24 | 14 | ADM, CAV2, CD33, CDH13, HMOX1, IGFBP6, OGN, RARRES1, CCL23, TPM1, CD300A, LDOC1, NOX4, HAVCR2 |
| GO BP | GO:0051051 negative regulation of transport | -6.18 | 11 | CD33, CRYAB, GEM, GJA1, NCKAP1L, HMOX1, MAP1B, PLN, UCP2, DYSF, CD300A |
| GO BP | GO:0031099 regeneration | -6.05 | 7 | ADM, GJA1, HMOX1, LCP1, MAP1B, UCP2, YAP1 |
| GO BP | GO:0061061 muscle structure development | -6.02 | 11 | ADM, CAV2, CDH2, CRYAB, CSRP2, FOXC1, TPM1, DYSF, SGCE, PDLIM3, NOX4 |
| GO CC | GO:0030667 secretory granule membrane | -5.82 | 9 | C5AR1, CAV2, CD14, CD33, FCER1G, NCKAP1L, PCDH7, PLAUR, CD300A |
| GO BP | GO:0010035 response to inorganic substance | -5.74 | 11 | AQP9, CD14, CRYAB, FABP4, HMOX1, MAP1B, MAPK13, S100A8, UCP2, ASPN, FIBIN |
| GO BP | GO:0048514 blood vessel morphogenesis | -5.67 | 10 | ADM, CALD1, CDH2, CDH13, COL8A1, CSPG4, FOXC1, GJA1, HMOX1, YAP1 |
| GO BP | GO:0097435 supramolecular fiber organization | -5.67 | 11 | CALD1, CSRP2, EXT1, FOXC1, FMOD, LCP1, PLS3, RAC2, TPM1, FBLN5, ENAH |
| GO BP | GO:0030155 regulation of cell adhesion | -5.61 | 13 | CDH13, COL8A1, COL16A1, CTSG, ECM2, NCKAP1L, PLAUR, RAC2, TPM1, FERMT2, CD300A, LIMCH1, HAVCR2 |
| GO BP | GO:0009611 response to wounding | -5.57 | 10 | ADM, CTSG, EXT1, HMOX1, MAP1B, PLAUR, S100A8, TPM1, DYSF, YAP1 |

GO, Gene Ontology; KEGG, Kyoto Encyclopedia of Genes and Genomes; CC, Cellular compartment; BP, Biological processes; MF, Molecular function.
